# Tamoxifen and its metabolites induce mitochondrial membrane depolarization and caspase‐3 activation in equine neutrophils

**DOI:** 10.1002/vms3.316

**Published:** 2020-06-17

**Authors:** Alejandro Albornoz, Natalia Morales, Benjamin Uberti, Claudio Henriquez, Rafael A. Burgos, Pablo Alarcon, Gabriel Moran

**Affiliations:** ^1^ Department of Pharmacology Faculty of Veterinary Sciences Universidad Austral de Chile Valdivia Chile; ^2^ Graduate School, Faculty of Veterinary Sciences Universidad Austral de Chile Valdivia Chile; ^3^ Department of Clinical Veterinary Sciences Faculty of Veterinary Sciences Universidad Austral de Chile Valdivia Chile

**Keywords:** apoptosis, horses, neutrophils, Tamoxifen

## Abstract

Neutrophils participate in innate immunity as the first line of host defence against microorganisms. However, persistent neutrophil activity and delayed apoptosis can be harmful to surrounding tissues; this problem occurs in diverse inflammatory diseases, including asthma‐affected horses. Previous studies in horses with acute lung inflammation indicated that treatment with tamoxifen (TX), a selective oestrogen receptor modulator, produces a significant decrease in bronchoalveolar lavage fluid (BALF) neutrophil content. The aim of this study was to investigate the effect of tamoxifen and its metabolites (N‐desmethyltamoxifen and endoxifen) on the mitochondrial membrane potential assay by flow cytometry, and the activation of effector caspase‐3 through immunoblotting, in peripheral blood neutrophils obtained from healthy horses (*n* = 5). Results show that tamoxifen, N‐desmethyltamoxifen and endoxifen depolarize the mitochondrial membrane and activate caspase‐3 in healthy equine neutrophils in vitro. These findings suggest that tamoxifen and its metabolites may activate the intrinsic apoptotic pathway in equine neutrophils. However, more studies are necessary to further explore the signalling pathways of these drugs in the induction of apoptosis.

## Graphical abstract

These findings suggest that tamoxifen and its metabolites can activate the intrinsic apoptotic pathway in equine neutrophils.

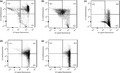

## 1 INTRODUCTION

Neutrophils are the final effector cells of innate immunity, with a primary role in the clearance of extracellular pathogens. Neutrophils are characterized by their short lifespan, as they survive for less than 24 hr in the bloodstream and are inherently pre‐programmed to die by constitutive apoptosis, with this turnover increasing during an infection (McCracken & Allen, 2014; Teng, Ji, Ji, & Li, 2017). Certainly, neutralization of the offending insult is essential for the resolution of inflammation; however, because of their potential to release large amounts of histotoxic and other pro‐inflammatory agents, excessive or dysregulated neutrophil responses and inadequate repair contribute to persisting tissue damage that underlies many inflammatory diseases (El Kebir & Filep, 2010).

Equine asthma is an immune‐mediated condition that develops in mature horses following stabling and inhalation of hay and straw dust, among other allergens (Robinson et al., 2010; Uberti & Morán, 2018). The disease is characterized by pulmonary neutrophilia and excessive mucus production, resulting in reduced dynamic lung compliance and increased pulmonary resistance and pleural pressure excursions (Jackson, Berney, Jefcoat, & Robinson, 2000). This disease is closely related to severe human asthma, which is characterized by the presence of neutrophils in the airways. In this pathology, the presence of prosurvival factors has been detected in bronchoalevolar fluid (BALF) from affected patients (Bruijnzeel, Uddin, & Koenderman, 2015). These same authors suggest that the delay in the rate of neutrophilic apoptosis seems to play an important role in the development of the disease, with neutrophils ultimately responsible for the maintenance and exacerbation stages of the disease. Considering the above, treatments that aim to regulate neutrophil homeostasis should be effective for this type of pathology.

Tamoxifen (TX) is a synthetic non‐steroidal anti‐oestrogen agent that is widely used for treating all stages of breast cancer and has been approved for the prevention of breast cancer in high‐risk women (Budtz, 1999; Cameron et al., 2000). Our research group has previously shown that TX increases in vitro early apoptosis of granulocytic cells from horse peripheral blood and BALF (Sarmiento et al., 2013). Our data also suggest that TX has an inhibitory effect on the kinesis of equine peripheral blood neutrophils stimulated with IL‐8 (Morales, Henriquez, Sarmiento, Uberti, & Moran, 2018). In addition, our data also showed that TX has the ability to induce in vivo apoptosis of granulocytic cells in a model of induced acute lung inflammation in horses, with concomitant clinical improvement (Perez et al., 2016). An independent group also found that TX produces an improvement in airway resistance in horses with exacerbated severe equine asthma, although no significant improvement in BALF neutrophil counts was observed in that study (Mainguy‐Seers, Picotte, & Lavoie, 2018). Thus, no conclusions should be made regarding the clinical applicability of TX until further data are available. To date, the mechanisms through which TX regulates apoptosis in horse neutrophils remain unknown.

Caspases are crucial for the initiation, propagation and execution of apoptosis. They are activated through two main pathways: the extrinsic or death receptor pathway and the intrinsic or mitochondrial pathway. The extrinsic pathway monitors the extracellular microenvironment, and it is induced by pro‐apoptotic stimuli recognized through specific cell surface receptors (e.g. TNFR or Fas) that lead to the activation of initiator caspases 8/10, and which subsequently activate effector caspases 3/6/7. The intrinsic pathway monitors the intracellular microenvironment, and it is induced by pro‐apoptotic stimuli such as damaged DNA or oxidative stress, resulting in the permeabilization of the outer mitochondrial membrane and the release of cytochrome c to the cytosol; together with Apaf‐1 and pro‐caspase‐9, they create a protein complex known as the apoptosome, which ultimately results in the activation of caspase‐3 (Degterev, Boyce, & Yuan, 2003; Galluzzi, Kepp, Trojel‐Hansen, & Kroemer, 2012). In certain cell types, the extrinsic pathway, initiated by cell surface receptors such as Fas, can cross‐talk with the intrinsic pathway (Wang & Youle, 2009).

Considering the potential use of TX in the treatment of equine asthma, it is necessary to expand our knowledge about the molecular mechanisms involved in the beneficial effect exerted by this drug. For this reason, the aim of this study was to evaluate the effect of TX and its active metabolites N‐desmethyltamoxifen and endoxifen on the depolarization of the mitochondrial membrane and the activation of effector caspase‐3.

## 2 MATERIALS AND METHODS

### 2.1 Horses

Five clinically healthy mixed‐breed adult horses (four mares and one gelding, body weight 420–450 kg, age 8–12 years) belonging to and housed at the Universidad Austral de Chile veterinary teaching hospital were enrolled in this study. All animals were housed on pasture and fed grass with free access to water. Physical examinations were performed before sample collection by qualified veterinarians to ensure that the animals were healthy. Each horse was sampled once, and each sample was analysed independently. All procedures were approved by the Universidad Austral de Chile Bioethics Committee for the Use of Animals in Biomedical Research (approval resolution no 251/2016).

### 2.2 Neutrophil isolation

The isolation of blood leukocytes was performed as previously described by our group (Borlone et al., 2017; Perez et al., 2016). Briefly, 10 ml of blood obtained by jugular venipuncture was placed in sterile tubes containing 1 ml of 3.8% w/v trisodium citrate. Blood was placed on a discontinuous density gradient (Percoll®; GE Healthcare), with 4 ml of 85% Percoll in the bottom of a 15 ml tube and 4 ml of 70% Percoll above. After centrifugation (45 min, 670 g), the upper layer contained mononuclear cells, and the lower layer contained granulocytes. Both layers were aspirated for further processing. Granulocyte cell purity and viability were assessed by flow cytometry (BD FACS Canto II). Viability was assessed using an Annexin V assay as previously described by our group (Perez et al., 2016). Cells were subsequently prepared for bioassays.

### 2.3 Mitochondrial membrane potential assay

The mitochondrial membrane potential was detected using the commercial MitoProbe™ JC‐1 Assay Kit. Briefly, 1 × 10^6^ neutrophils were treated with TX and its metabolites (N‐desmethyltamoxifen and endoxifen) at concentrations of 10 µM and 15 µM or with vehicle (DMSO 0.2%) and incubated for 30 min at 37°C. After treatment with TX and its metabolites, the cells were pelleted and resuspended in 250 µl of HBSS buffer and then stained with 2 µM JC‐1 for 20 min at 37°C. Cells were washed with HBSS without calcium and resuspended in 250 µl of HBSS for analysis with flow cytometry using 488 nm excitation with 530/30 nm and 585/42 nm bandpass emission filters. CCCP, a mitochondrial membrane potential disrupter, was used as a positive control. The results of the mitochondrial membrane potential assay were measured by the ratio of the mean fluorescence intensity (MFI) of PE‐A/FITC‐A. The JC‐1 assay forms red aggregates in healthy and intact mitochondria, and green monomers in the cytoplasm when mitochondrial membrane integrity is compromised.

### 2.4 Immunoblotting

Neutrophils (5 × 10^6^) were treated with 10 µM TX and its metabolites (N‐desmethyltamoxifen and endoxifen) or vehicle (DMSO 0.2%) and then incubated at 37°C for 0, 5, 10, 20, 30 and 60 min. The cells were lysed, and 60 µg of purified protein was analysed by 12% SDS‐page and transferred to a nitrocellulose membrane. The primary antibody against procaspase and caspase‐3 (Cell Signaling) was incubated for 1 hr. The membranes were developed using Super Signal West Femto (Thermo Scientific) and visualized on the Odyssey Fc Imaging System. Each membrane was re‐probed with anti‐vinculin antibody (Santa Cruz).

### 2.5 Statistical analysis

GraphPad Prism (GraphPad Software Inc., version 5.0) was used for graph generation and statistical analysis. Kolmogorov–Smirnov tests showed that the data were normally distributed for all mitochondrial membrane potential assays. ANOVA was performed to compare the differences between groups in each assay. When significant, means were compared using Tukey's Multiple Comparison Test. Results are reported as mean ± *SD*. A value of *p* < .05 was considered significant.

## 3 RESULTS

**Figure 1:**
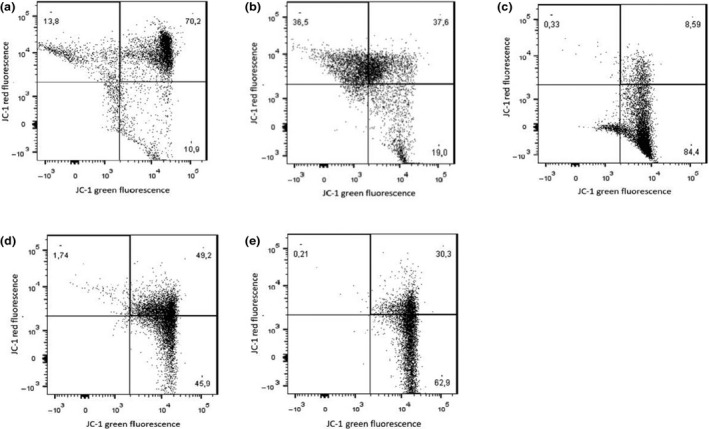
Representative flow cytometry histograms showing JC‐1 staining after treatment with different concentrations of tamoxifen. (a) Untreated cells stained with JC‐1, (b) cells with DMSO 0,2 %, (c) positive control with CCCP (5 µM), (d) cells treated with 10µM of tamoxifen, (e) cells treated with 15 µM of tamoxifen.

**Figure 2:**
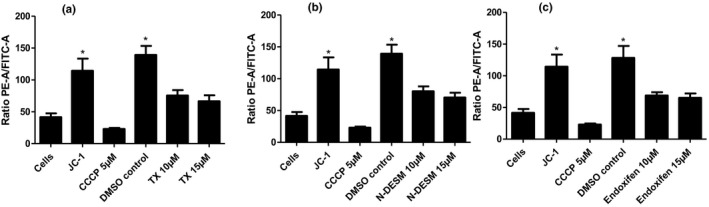
Mitochondrial membrane potential representative results for basal cell incubation without markers, untreated cells marked with JC‐1, cells marked with CCCP, drug vehicle control (DMSO 0.2%), and incubation with tamoxifen (a), N‐desmethyltamoxifen (b) and endoxifen (c). Results are shown as the mean ratio between red fluorescence and green fluorescence for each condition indicated. Values are represented as mean + SD of five independent experiments (*p < .05 between different conditions).

Mitochondrial membrane potential representative flow cytometry results are presented in Figures 1a–e, and 2 shows the statistical representation of these histograms, in which TX treatment showed a statistically significant effect on the polarization of the mitochondrial membrane compared to the negative control (DMSO 0.2%) at 10 and 15 µM (*p* < .05). Treatment with N‐desmethyltamoxifen showed significant differences compared to the negative control at 10 and 15 µM (*p* < .05). Treatment with endoxifen showed a significant effect compared to the negative control (DMSO 0.2%) at 10 and 15 µM (*p* < .05).

**Figure 3:**
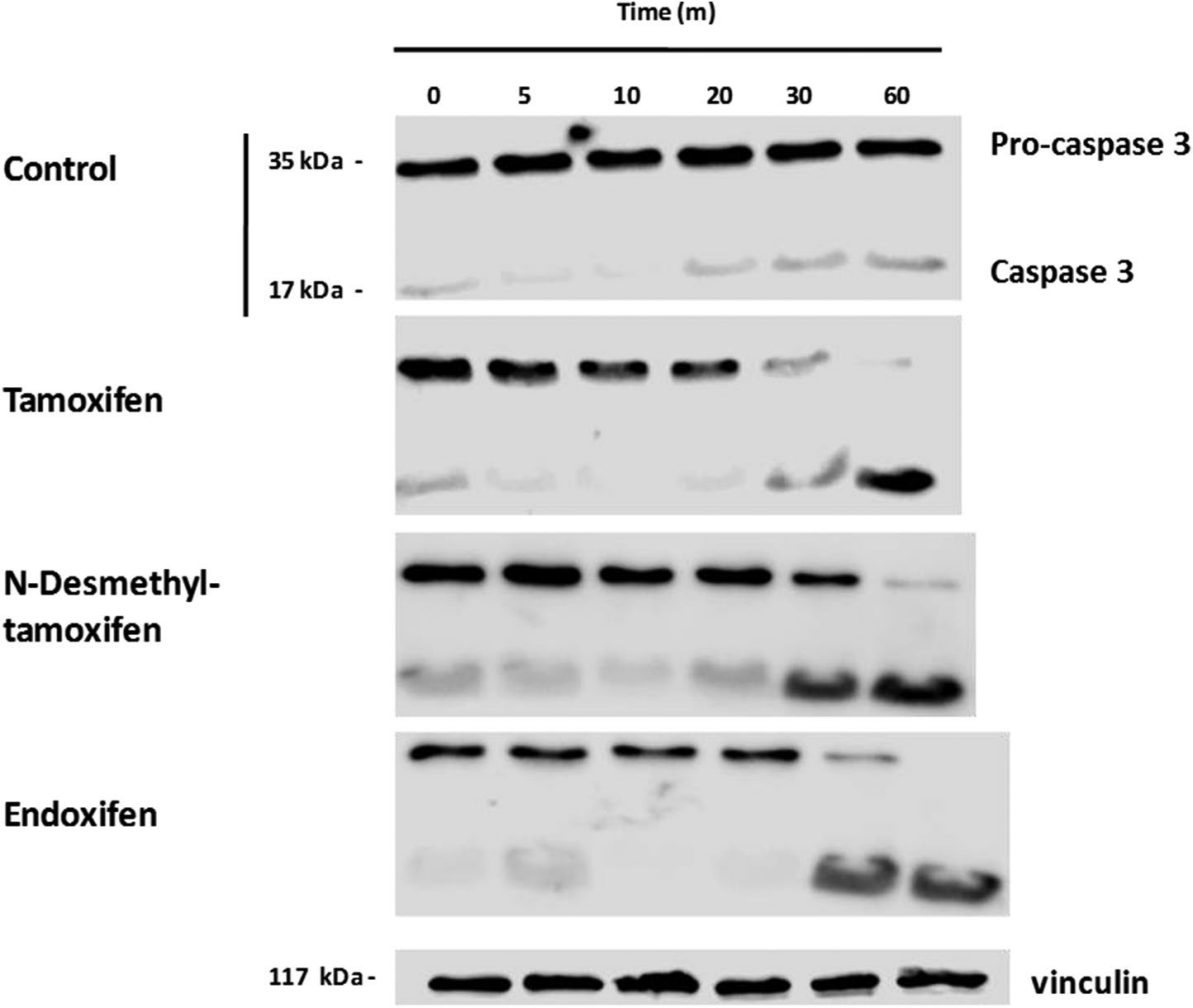
Immunodetection of caspase 3 and pro‐caspase 3 in equine neutrophils treated with tamoxifen, N‐desmethyltamoxifen, endoxifen or vehicle (DMSO 0,2 %) at 0, 5, 10, 20, 30 and 60 min. The results shown are representative of results from 2 independent experiments.

The activation of apoptosis was also confirmed by western blot analysis. The activation kinetics of procaspase and caspase‐3 showed that TX and its two metabolites (10 µM) induce the activation of this caspase, and this effect was visibly evident after 30 min of incubation (Figure 3).

## 4 DISCUSSION

In this work, we show that like TX, the biologically active TX metabolites desmethyltamoxifen and endoxifen have a pro‐apoptotic effect on equine neutrophils, as shown by alteration of mitochondrial membrane potential and caspase‐3 activity. Although it is necessary to more deeply investigate the mechanisms used by these drugs in equine neutrophils, this data suggest that TX and its metabolites are capable of activating the intrinsic apoptotic pathway. In this study, it was observed that all of these agents are capable of activating caspase‐3, an effector caspase of the apoptotic pathway. It was also shown that desmethyltamoxifen and endoxifen promote the depolarization of the mitochondrial membrane. Certainly, mitochondria play a key role in caspase activation because the release of cytochrome c from the inner membrane of the mitochondria into the cytosol is a necessary step for the conformation of the apoptosome. The release of cytochrome c as an effect of chemotherapeutic drugs has been described previously (Emert‐Sedlak et al., 2005). Dietze, Caldwell, Grupin, Mancini, and Seewaldt (2001) showed direct evidence of the key role of this organelle in the apoptotic effect of TX, through in vitro experiments with mitochondria isolated from rat liver.

Our group has previously demonstrated in vitro that TX exerts its effect via a mechanism that is independent of its interaction with oestrogen receptors (ER) (Olave, Morales, et al., 2018). It has been shown that TX exerts many off‐target effects, and ultimately, it is possible that as a whole, these off‐target effects mediate the therapeutic effect of the drug (Mandlekar & Kong, 2001). Here, we determined that 30 min with TX is long enough to appreciate the depolarization of the mitochondrial membrane and the activation of caspase‐3. This result correlates with the anti‐inflammatory effects documented by our group (Borlone et al., 2017; Olave, Alvarez, et al., 2018; Olave, Morales, et al., 2018; Perez et al., 2016; Sarmiento et al., 2013). Paradoxically, a pro‐inflammatory effect for this drug has also been documented by other authors. TX was able to potentiate the bactericidal effect of human neutrophils, producing an increase in the production of NETs, after 2 hr of drug exposure (Corriden et al., 2015). These results suggest that the exposure time is an important factor in determining the effect of this drug.

In summary, although the molecular mechanism has not yet been fully elucidated, our data indicate that TX and its metabolites (N‐desmethyltamoxifen and endoxifen) are able to induce apoptotic pathways via a mechanism that involves alteration of mitochondrial membrane potential and caspase‐3 activation in equine neutrophils. As mentioned earlier, our group has shown the beneficial effect of this drug in the treatment of horses with inflammation of the airways. Considering the importance of correct neutrophil homeostasis in the development of equine asthma, the information collected in this study and ongoing work by our group will allow the design and evaluation of more effective treatments for this disease.
